# Supplementary material for: Diffusion models enable zero-shot pose estimation for lower-limb prosthetic users
Source: PLOS Digit Health. 2025 Mar 31;4(3):e0000745. doi: 10.1371/journal.pdig.0000745 (PMC11957558; doi:10.1371/journal.pdig.0000745)

Supplementary Figure 1. A schematic diagram representing the breakdown of videos for each type of amputation.

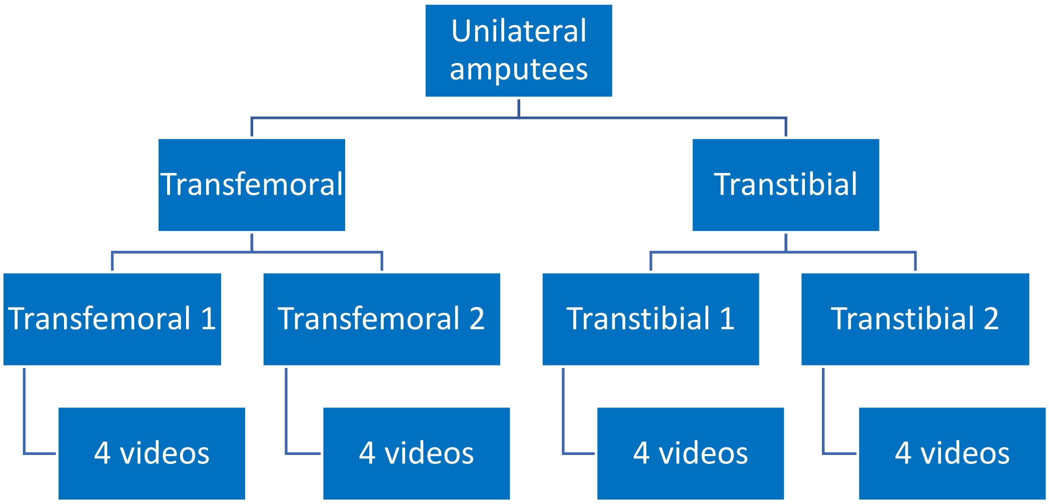

Supplement: S1 Fig — (PDF) [file pdig.0000745.s001.pdf]
