# Supplementary material for: Diffusion models enable zero-shot pose estimation for lower-limb prosthetic users
Source: PLOS Digit Health. 2025 Mar 31;4(3):e0000745. doi: 10.1371/journal.pdig.0000745 (PMC11957558; doi:10.1371/journal.pdig.0000745)
Supplement: Attachment — Submitted filename: ResponseToReviewers.docx [file pdig.0000745.s010.docx]

We thank all the reviewers for their comments and suggestions. We have made every effort to address and incorporate them. We have also attached the revised manuscript with and without tracked changes. To ease readability, the revised manuscript with track changes still follows the old format where the Methods is the last section. The revised manuscript without track changes is where the Methods section is moved after the Introduction section, following the reviewer’s advice.

Below is a point-by-point response to each reviewer’s comments.

Reviewer #1:

The paper titled "Diffusion models enable zero-shot pose estimation for lower-limb prosthetic users" presents a novel approach for improving pose estimation for lower-limb prosthetic users using generative diffusion models. This approach transforms images of prosthetic limbs into able-bodied representations, allowing standard pose estimation models to better detect key points without requiring additional data collection or model retraining.

The use of diffusion models to transform prosthetic limb images into able-bodied representations is a creative solution to a challenging problem. Additionally the method shows substantial improvements with reductions in coordinate errors of 37% for transtibial and 76% for transfemoral prosthetic limbs. However some minor modifications are suggested for the publication:

- The authors should verify clarity and writing, to be more concise, for example in introduction lines 43 - 50 are really similar to 51 - 59.

We have removed paragraph lines 44 – 60, following the advice of another reviewer as it was deemed out of scope.

- the method is clear in text, but improving the figure 6 and adding more details could help to clarify the methodology used.

We have taken note on the feedback and have made a few changes to Figure 6. Due to the change in the arrangement of Methods section, the previous Figure 6 is now Figure 1. We have also expanded the figure subtitle to make it clearer.

Changes in manuscript line 405 (tracked changes) and 105 (no tracked changes):

*“Fig 1. Workflow of the proposed zero-shot method. (A) The standard workflow for applying pose estimation (e.g., OpenPose) directly on the original image. In this example, OpenPose fails to accurately identify keypoints on the prosthetic limb, as highlighted by the red circle. (B) The proposed zero-shot workflow leverages ControlNet. The original image is processed using the Canny edge detector to generate an edge map, which is then passed through Stable Diffusion to create a synthetic image. This synthetic image is subsequently used for pose estimation. In this case, improvements in pose estimation accuracy, particularly in the identification of lower-limb keypoints, are evident, as highlighted in the red circle.”*

- More details on the edge map generation process and its potential limitations would be helpful. The authors should discuss how variations in edge map quality might affect the overall pose estimation accuracy.

We have included more details on the edge map generation process in the methods section and highlighted limitations in the discussion.

Changes in manuscript lines 452-462 (tracked changes) and lines 152-162 (no tracked changes):

*“Edge maps were generated using the Canny edge detector, which works by identifying areas of rapid intensity change, thus highlighting the boundaries of objects in the image [31]. It starts by applying a Gaussian filter to smooth the image, which helps reduce noise and makes the edges clearer. Next, it calculates the intensity gradients to identify areas where there is a rapid change in brightness, indicating potential edges. To determine which edges are significant, the detector uses two threshold values: a high threshold to mark strong edges and a low threshold to include weaker edges that are connected to these strong ones. This approach ensures that important edges are preserved while minimizing noise, resulting in a clear edge map that highlights the boundaries of objects in the image. These edge maps subsequently serve as the conditioning information for the diffusion model.”*

Changes in manuscript lines 265 to 276 (tracked changes) and lines 399-406 (no tracked changes):

*“The performance of our zero-shot method is inherently dependent on the quality of the input images and the resulting edge maps. From our experience, the Canny edge detector's effectiveness can be influenced by variations in lighting, texture, and noise levels in the original images. This variability can lead to several issues, such as low-contrast regions failing to produce well-defined edges, resulting in incomplete edge maps. Moreover, details like overlapping limbs, might not be adequately captured, causing ambiguities in the edge map. This limitation is particularly evident in our observation of increased swapping of lower-limb keypoints in the sagittal plane (S1 Table).”*

- The specific configuration and parameters used in ControlNet or to the data should be elaborated on, including any modifications made for this study.

We used the default configuration. The only change was made to the prompts where we have initially included the specific prompts and negative prompts used for all videos. We have included a statement to make it clear that the default configuration was used.

Changes in manuscript lines 465 to 467 (tracked changes) and lines 165-167 (no tracked changes):

*“We used the default configuration such as an annotator resolution of 512, a canny low threshold of 100, and a canny high threshold of 200.”*

- The use of publicly available videos from YouTube introduces variability in video quality and recording conditions. The paper should discuss the implications of these constraints on the generalizability of the results.
Thank you for the suggestion. We have included these implications as part of our limitation.

Changes in manuscript lines 349-358 (tracked changes) and lines 479-488 (no tracked changes):

*“The use of YouTube videos as our data source introduces its own set of limitations. These videos vary in quality and recording conditions can affect the performance of our method. Additionally, the calculation of the MAE involves an assumption about the subject's height, potentially introducing inaccuracies. While the results from these open-source videos are promising, practitioners aiming to replicate our method may benefit from controlled video recordings with known parameters, potentially leading to improved outcomes. For instance, optimizing background conditions could enhance edge detection quality and subsequent pose estimation. However, this study also demonstrates that despite uncontrolled recording conditions, valuable information can still be extracted, making the method applicable to existing videos.”*

- The study relies on DeepLabCut for ground truth keypoints, but further validation with more robust ground truth data would strengthen the findings. It would be beneficial to include more detailed comparisons with other ground truth datasets if available.

We acknowledge the limitation of using DeepLabCut as ground truth keypoints and have added in our manuscript.

Changes in manuscript lines 340-348 (tracked changes) and lines 470-478 (no tracked changes):

*“It is important to note that our ground truth keypoints were obtained using DeepLabCut. While DeepLabCut is a powerful tool for pose estimation, it may introduce some uncertainties and potential errors. To enhance the robustness of our findings, further validation using traditional marker-based motion capture systems would be beneficial. Comparing the results of our zero-shot method with data from a marker-based system would provide a more comprehensive evaluation of its performance. This additional validation step could strengthen the credibility of our approach and offer valuable insights into any discrepancies between markerless and marker-based techniques, particularly in the context of prosthetic gait analysis.*

- The results section is thorough, but could benefit from a clearer separation of key findings and their implications. Highlighting the most significant results in a summary table or bullet points could enhance clarity.

Thank you for the suggestion. We have initially included a brief key findings in the introduction. We have moved the paragraph to the Discussion section and included the implication of our work.

Changes in manuscript lines 200-213 (tracked changes) and lines 334-347 (no tracked changes):

*“To our knowledge, this is the first work that achieves zero-shot pose estimation for lower-limb prosthetic users. The contributions of this paper are twofold. First, we provide quantification of the errors made by one of the most widely used pre-trained pose estimation software, OpenPose, on lower-limb prosthetic users. Second, we demonstrate a working method for accurate zero-shot pose estimation on lower-limb prosthetic users without the need for any data collection, labelling or training. Our zero-shot method achieved 37% to 76% decrease in error for pose estimation on transtibial and transfemoral prosthetic limbs respectively. This work serve to demonstrate the feasibility of the zero-shot approach which is a step towards advancing personalized rehabilitation and improving quality of life outcomes through gait analysis for the lower-limb amputee population. By improving gait analysis for the lower-limb amputee population, our approach has the potential to enhance the development of custom rehabilitation strategies, ultimately improving mobility and overall quality of life outcomes for prosthetic users.”*

Reviewer #2:

This study by Zhou et al. presents a new method for video-based gait analysis in prothetic users that corrects errors in keypoint-tracking of the prosthetic limb. This is important because a conventional and pre-trained pose estimation algorithm like OpenPose does not track the prosthetic limb well. The authors find that this new “zero-shot” method improves lower-limb keypoint tracking of the prosthetic limb compared with OpenPose.

There is no doubt that this paper addresses an important issue: video-based gait analysis of prosthetic gait is an important topic and that there are challenges with correctly tracking the movements of the prosthetic limb. However, I have concern about the limited data set used for validation in the study. Considering that only 4 unique gait trials were used of 2 individuals – I think the manuscript should be considered a preliminary report which must be clearly highligted.

Major Comments

1) The data set only comprises 2 individuals and only 4 unique gait trials (captured by 4 cameras giving a total of 16 videos). Furthermore, it appears that only the sagittal videos were used which would bring the number of videos down to 8. And last, only the limb closest to the camera was analyzed. From an already limited data set, this whittles down the data set significantly.

While I have no doubt that the “zero-shot” method dramatically improves keypoint tracking relative to OpenPose – this paper is really more of a preliminary report. I’d like the authors to consider sourcing additional videos for this paper. Alternatively, data limitation must be highlighted in the abstract, results, and conclusion.

Indeed the data only consist of 2 individuals. We apologize for the oversight but have included the results of trunk lean.

We have taken your suggestion and included the limitation in the abstract and discussion sections.

Changes in manuscript Abstract:
*“While the results demonstrate the feasibility of markerless gait analysis for lower-limb prosthetic users, the study's findings are based on a limited dataset of publicly available videos, and caution should be exercised in generalizing the results to broader populations due to the varying nature of prosthetic designs.”*

Changes in manuscript lines 359-366 (tracked changes) and lines 489-496 (no tracked changes):

*“In addition to these technical limitations, it is crucial to acknowledge the constraints of our data set. We assessed the proposed method on only two individuals. As such, our results should be considered as a preliminary report and proof of concept, demonstrating that the zero-shot method is a potential solution for obtaining kinematics data from lower-limb amputees. The zero-shot performance may vary across a broader population with diverse prosthetic designs and individual characteristics. Further research with a larger and more diverse sample size is necessary to validate the robustness and wide applicability of this zero-shot method.”*

2) I’d like the authors to reflect on the reliability of the ground truth keypoint tracking using DLC. First, how are keypoints defined at the “knee”, “ankle” and “toes” in a prosthetic limb? Second, the authors report an MAE of 8 pixels using DLC (line 411). This translates to an error of ~2.4 cm (using conversion on line 140). Is 2.4 cm within an acceptable limit of joint location in a prosthetic limb?

The prosthesis-side markers were aligned with intact-side markers as closely as possible when anatomical landmarks were inaccessible or absent. This is similar to other studies on transtibial (Orekhov et al., 2019) and transfemoral (Hood et al., 2020). We have included this information in the manuscript.

The use of markerless pose estimation on lower limb amputees is scarce. Hence, we have used a proxy on able-bodied limbs accuracy where we have provided the joint and angle error in S4 Table. Given that 2.4cm is within the range of studies that have used multi-camera on able-bodied limbs, we postulate that 2.4cm would be a fairly acceptable accuracy of joint location in a prosthetic limb.

We have also included a limitation to highlight the use of DLC as ground truth.

Changes in manuscript lines 497-500 (tracked changes) and lines 197-200 (no tracked changes):

“The prosthesis-side keypoints were aligned with intact-side keypoints as closely as possible when anatomical landmarks were inaccessible or absent, similar to other studies on transtibial [35] and transfemoral [36] amputees.”

Changes in manuscript lines 340-348 (tracked changes) and lines 470-478 (no tracked changes):

*“It is important to note that our ground truth keypoints were obtained using DeepLabCut. While DeepLabCut is a powerful tool for pose estimation, it may introduce some uncertainties and potential errors. To enhance the robustness of our findings, further validation using traditional marker-based motion capture systems would be beneficial. Comparing the results of our zero-shot method with data from a marker-based system would provide a more comprehensive evaluation of its performance. This additional validation step could strengthen the credibility of our approach and offer valuable insights into any discrepancies between markerless and marker-based techniques, particularly in the context of prosthetic gait analysis.”*

References:

Orekhov, G., Robinson, A. M., Hazelwood, S. J., & Klisch, S. M. (2019). Knee joint biomechanics in transtibial amputees in gait, cycling, and elliptical training. Plos one, 14(12), e0226060. https://doi.org/10.1371/journal.pone.0226060

Hood, S., Ishmael, M. K., Gunnell, A., Foreman, K. B., & Lenzi, T. (2020). A kinematic and kinetic dataset of 18 above-knee amputees walking at various speeds. Scientific data, 7(1), 150. https://doi.org/10.1038/s41597-020-0494-7

Minor Comments

1) Introduction: The intro is quite long and I can it will be improved by omitting the section on observational gait analysis which is outside the scope of the paper.

We have removed the paragraphs on observational gait analysis.

2) Table 1: Why are MAE of prosthetic transfemoral joint coordinates using OpenPose reported as >100?

The error value is considerably large due to a large number of frames removed consecutively during data processing for certain anatomical landmarks. Hence, to ease readability, we decided to have the value truncated. Interested readers can still refer to S2 Table which contains the exact error value for each anatomical landmark.

3) Methods, line 419: How many frames with likelihood less than 0.50 were removed? Please report in manuscript.

We have included it as supplementary table S7 and referenced it accordingly in the manuscript text.

Changes in manuscript lines 514-515 (tracked changes) and lines 214-215 (no tracked changes):

*“Consequently, frames with a likelihood below 0.50 were removed (S7 Table).*”

4) Methods, lines 437-441: lateral trunk lean is described here, but is not reported in the Results.

We apologize for the oversight. We have now included lateral trunk lean results and discussed its results in the Discussion section.

Changes in manuscript lines 536-538 (tracked changes) and lines 236-367 (no tracked changes):

*“To ensure consistency with existing literature, we calculated the lateral trunk lean angle in both abled and prosthetic limb gait cycles.”*

Changes in manuscript lines 194-198 (tracked changes) and lines 327-332 (no tracked changes):

*“Similarly, the lateral trunk lean angle was analyzed from the anterior camera view. Although error quantification was not possible due to the absence of ground truth labels, both amputees exhibited a positive lean toward the prosthetic limb during the stance phase (Fig. 5). The kinematic curves generated by OpenPose and the zero-shot method were quite similar.”*

Changes in manuscript lines 237-249 (tracked changes) and lines 371-383 (no tracked changes):

*“In the anterior camera view, lateral trunk lean is a common compensatory strategy observed among lower-limb amputees [38-41]. Consistent with findings in existing literature [39,41] our analysis also revealed that both transtibial and transfemoral subjects leaned toward the prosthetic limb during the stance phase. When comparing the kinematic curves generated by OpenPose and our zero-shot method, we found little differences between them. This may suggest that current pose estimation methods may already offer sufficient accuracy for frontal-plane analysis. This is unsurprising, as the calculation of lateral trunk lean angles does not account for the presence of the prosthetic limb. Thus, altering a prosthetic limb to resemble an able-bodied limb in our zero-shot method has minimal impact on the resulting kinematic outcomes. Practitioners may opt to use pose estimation algorithms directly to quantify lateral trunk lean angles, though the accuracy of these measurements remains to be thoroughly validated.”*

5) Figures 2-4: The is repetition across the figures as curves are duplicated between 2 and 3 and between 3 and 4. I strongly suggest that the authors either collapse into a single figure of many subplots or divide into 2 figures.

Thank you for the suggestion. After careful consideration, we have decided to remove the old Figure 3 to avoid redundancy, while retaining the old Figures 2 and 4. These figures present results for transtibial (now Figure 3) and transfemoral (now Figure 4) pose estimation separately, which we believe provides clearer insight into the improvements achieved by the zero-shot method for each case. The old Figure 3 primarily compared transtibial and transfemoral results, and we agree that this content overlaps with what is already conveyed in the old Figures 2 and 4. We hope this adjustment will streamline the presentation while still allowing readers to fully appreciate the improvements in pose estimation for both groups.

6) Figures 5 and 6: the image quality is very poor.

Thank you for the comment. We have re-created Figures 5 and 6 and exported them in tiff format. The old Figure 5 is now Figure 6 and the old Figure 6 is now Figure 1 in the revised manuscript, after re-arranging the order of the Methods section.

Reviewer #3:

This manuscript meets the PLOS Digital Health’s publication criteria in terms of:
1. Originality - a novel zero-shot pose estimation method to achieve accurate pose estimation for lower limb prosthetic users is presented;
2. High Importance and broad interest - the significant problem of markerless pose estimation models in accurate localization of joints on prosthetic limbs for quantitative gait analysis is outermost important for assessing and rehabilitating lower-limb amputee population;
3. High methodological rigor and ethical standards - methods are robust and appropriate for the research question and ethical standards are maintained since publicly accessible videos from a non-profit organization were used;
4. Substantial evidence for its conclusions - conclusions are supported by convincing results following a thorough and appropriate analysis, complemented with supplementary material;
5. Clearly outlined utility and accessibility - findings are straightforward presented to be easily understood and utilized by the broader digital health community;
6. Appropriate standards and practice of open science - the manuscript adhere to principles of transparency reproducibility (methods described in detail), and data sharing (data available in the supplementary material and reference to the GitHub code for the adopted model).
The methods used for statistical evaluation are clearly described, and appropriate techniques (with reference to current literature methods) have been employed to ensure the robustness and validity of the results. Statistical tests on the comparison (e.g., in terms of normalized joint angle kinematics curves) of the zero-shot pose estimation method to ground truth and standard pose estimation models (i.e., OpenPose) can be added to enrich the statistical analysis.
As the authors mention, the raw data supporting the conclusions of the manuscript need to be made available.
The text is clear and well-structured, allowing readers to easily understand the research objectives, methods, results, and conclusions. However, it’s advised to move the Method section before the Results paragraph for a more comprehensive outline of the study’s findings.
The use of standard English throughout the manuscript ensures that it is accessible to a broad audience.
Finally, to further improve the manuscript, comments in the document uploaded by the reviewer should be addressed.

Thank you for your feedback.

We have moved the method section before the results paragraph.

Given that sample size we have, we did not perform any statistical comparison.

We have also included the raw data supporting the conclusions of the manuscript.

We have also addressed your comments indicated in the document.

Below are the changes made to the manuscript based on the comments in the document:

This paragraph (line 51-59) is a repetition of the previous one (line 43-50). Please choose only one formulation.

We have removed paragraph lines 44 – 60, following the advice of another reviewer as it was deemed out of scope.

Methods:

Dataset:

Please explicate the difference between transfemural1 and transfemural2 (first subject, female), and, similarly, between transtibial1 and transtibial2 (second subject, male).

We have included the differences in the manuscript.

Changes in manuscript lines 384-387 (tracked changes) and lines 84-87 (no tracked changes):

*“For the transfemoral subject, the same prosthesis was used across all recordings. In contrast, the transtibial subject used two different types of prostheses: a running blade in Transtibial 1 and a prosthesis resembling a conventional lower limb in Transtibial 2.”*

Results:

Please refer to Figure 3 also for the hip and ankle angle.

We have referred the sentence to Figure 3.

Changes in manuscript lines 158-160 (tracked changes) and lines 299-301 (no tracked changes):

“The hip angle curve exhibited satisfactory results for both transtibial (Fig. 2) and transfemoral (Fig. 3) amputees when employing both the OpenPose and zero-shot methods.”

Discussion - Limitations paragraph:

First sentence: The increased swapping is clearer in Table S1 than in Figure 1. Please consider to refer to Table S1.

We have referred the sentence to S1 Table.

Changes in manuscript lines 272-276 (tracked changes) and lines 405-407 (no tracked changes):

*“This limitation is particularly evident in our observation of increased swapping of lower-limb keypoints in the sagittal plane (S1 Table).”*

Fig. 5 provides an illustrative example of the observed phenomenon.
*Refer to Fig. 5A instead of Fig. 5, differentiating subfigures like in the following sentences of the paragraph.*

The old Figure 5 is now Figure 6. We have referred the sentence to Fig. 6A.

Changes in manuscript lines 282-283 (tracked changes) and lines 412-413 (no tracked changes):

*“Fig. 6A provides an illustrative example of the observed phenomenon.”*

“Moreover, the zero-shot method resulted in the reduction of frames where keypoints were not detected in OpenPose due to the non-resemblance of intact limbs.”

*Please refer again to Table S1.*

We have referred the sentence to S1 Table.

Changes in manuscript lines 289-291 (tracked changes) and lines 419-421 (no tracked changes):

*“Moreover, the zero-shot method resulted in the reduction of frames where keypoints were not detected in OpenPose due to the non-resemblance of intact limbs (S1 Table).”*

Assumption of the subject's height can also be discussed.

We have discussed the assumption of using the subject’s height in our limitation.

Changes in manuscript lines 351-352 (tracked changes) and lines 481-482 (no tracked changes):

*“Additionally, the calculation of the MAE involves an assumption about the subject's height, potentially introducing inaccuracies.”*
